# Figure disembedding facility and reduced left visual field bias are linked to the social dimension of autistic traits

**DOI:** 10.3758/s13414-025-03105-7

**Published:** 2025-06-09

**Authors:** Michael C. W. English, Isabelle M. Raiter, Nigel T. M. Chen, Diana W. Tan, Fabrice B. R. Parmentier, Troy A. W. Visser, Murray T. Maybery

**Affiliations:** 1 School of Psychological Science, University of Western Australia, Perth, WA Australia; 2 School of Population Health, Curtin University, Perth, WA Australia; 3 Macquarie School of Education, Macquarie University, Macquarie Park, NSW Australia; 4 Department of Psychology and Research Institute for Health Sciences, University of the Balearic Islands, Palma, BI Spain

**Keywords:** Autism, Autistic traits, Left visual field bias, Greyscales, Embedded figures, Hemisphere dominance

## Abstract

In separate lines of work, facility in detail-focused local processing and reduced left visual field (LVF) bias have been associated with autism. Plausibly, local-processing facility and reduced LVF bias could reflect a common neurocognitive mechanism – most likely reduced right-hemisphere dominance in visual attention. To test this possibility, undergraduate students selected to differ systematically in social and non-social autistic traits completed tasks assessing local-processing facility (Leuven Embedded Figures) and LVF bias (greyscales task). Participants with more pronounced social difficulties showed greater local-processing ability and reduced LVF bias compared to participants with less pronounced social difficulties. Local-processing ability also correlated negatively with LVF bias. This is the first study to examine both LVF bias and local-processing preference in the context of autism. The finding of relationship between these two cognitive features is behavioural evidence supporting the notion of a common underlying neurocognitive mechanism and of potential alterations in right hemisphere activation as a function of autism.

**Supplementary Information:**

The online version contains supplementary material available at 10.3758/s13414-025-03105-7.

## Introduction

With autism presenting as difficulties in social communication and restricted and repetitive behaviours and interests, considerable effort has been invested in identifying atypical cognition associated with these distinctive behaviours (e.g., see Happé & Frith, 2006; Pellicano & Burr, 2012; Velikonja et al., 2019). One form of atypicality in visuospatial cognition is that autism is associated with greater proficiency in, or reliance on, local processing (i.e., preferentially processing ‘details’ over ‘global structure’). While there has been debate as to whether enhanced local processing is accompanied by limited global processing, numerous studies have reported a local processing advantage for autistic compared to non-autistic individuals (Koldewyn et al., 2013; Mottron et al., 2003; Plaisted et al., 1999; Rinehart et al., 2000; Wang et al., 2007) and also for individuals with high (but not necessarily clinical) levels of autistic traits compared to individuals with low trait levels (e.g., see Almeida et al., 2010; Grinter et al., 2009a, b). This superior local processing associated with autism has been demonstrated using embedded figures tasks, which require locating simple shapes embedded in more complex visual displays. Meta-analyses have established that autistic individuals and those with high levels of autistic traits locate the hidden shapes faster and more accurately than non-autistic individuals and those with low levels of autistic traits (Cribb et al., 2016; Muth et al., 2014).

**Fig. 1:**
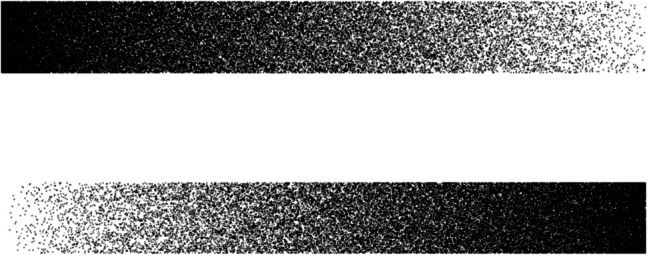
An example of stimuli of the greyscales task

Significantly, there is also evidence that autism is characterised by reduced asymmetry in visuospatial attention across the left and right visual fields (see English et al., 2023, for a review). While most people attend preferentially to information in the left visual field (LVF) (Jewell & McCourt, 2000; Nicholls et al., 1999), with this bias argued to reflect asymmetric cortical organisation and right-hemisphere specialisation for visuospatial processing (Davidson & Hugdahl, 1996; Foxe et al., 2003), researchers have demonstrated that autistic people show a reduced (or absent) LVF bias relative to non-autistic people, using various behavioural paradigms (Drmic, 2007). Reduced LVF bias has also been demonstrated for individuals with high levels of autistic traits compared to individuals with low trait levels (English et al., 2015, 2017). One task used to investigate LVF bias is the greyscales task in which the participant decides which of two horizontal bars – one shaded white-to-black and the other black-to-white – is the darker (see Fig. 1). Individuals not on the autism spectrum favour selecting the bar shaded darker on the left, consistent with enhanced attention to the LVF, whereas this LVF bias is reduced (or absent) for individuals with either a diagnosis of autism (Drmic, 2007) or with high levels of autistic traits (English et al., 2015, 2017).

With autism linked to greater proficiency in local processing and to reduced LVF bias in the visuospatial domain, could a common neural mechanism contribute to these two characteristics? One possibility is that both characteristics reflect underlying differences in hemispheric asymmetry. As mentioned above, LVF bias in visuospatial attention is thought to be a ‘side-effect’ of asymmetric cortical organisation such that visuospatial processing is predominantly a function of the right hemisphere (Fierro et al., 2000; Fink et al., 2002; Foxe et al., 2003; Siman-Tov et al., 2007). Significantly, hemispheric asymmetry has also been proposed for local and global processing. Broadly speaking, global processing is associated with greater right (relative to left) hemisphere activation whilst local processing is associated with greater left (relative to right) hemisphere activation (Flevaris et al., 2010; Hübner & Studer, 2009; Iglesias-Fuster et al., 2014; Ivry & Robertson, 1998; Weissman & Woldorff, 2005). Taken together, these two bodies of literature suggest that reduced right hemisphere dominance in visuospatial processing could underlie both a facility in or preference for local over global processing and reduced LVF bias in visuospatial attention in autism.

While investigating cognitive mechanisms and cortical functioning in autistic and non-autistic groups (or high and low autistic-trait groups) has merit, it is now recognised that autism is highly heterogenous. In particular, there is substantial evidence that the two major dimensions of autistic symptoms or traits – social communication difficulties and restricted and repetitive behaviours and interests – are dissociable in both behaviour (English et al., 2020; Fountain et al., 2012; Hurley et al., 2007; Lord et al., 2015; Mandy & Skuse, 2008; Pickles et al., 2014; Shuster et al., 2014) and heritability (Geschwind & State, 2015; Ronald et al., 2006; Warrier et al., 2019). The upshot of this dissociability is that a more nuanced approach to investigating cognitive mechanisms is needed; differences in cognition should be explored as a function of dimensions of autistic symptoms or traits rather than for autism conceptualized solely as an amalgam of dimensions.

This is all the more important here because both local processing and LVF bias have been associated with social, but not non-social, dimensions of autistic symptoms or traits. First, for a sample of autistic children, Pellicano et al. (2006) reported that faster times to locate shapes on the children’s embedded figures test (EFT) were associated with more pronounced difficulties in social interaction, rather than with difficulties in communication or restricted and repetitive behaviours and interests. Second, Russell-Smith et al. (2012) compared groups of adults selected for high and low levels of traits on each of two factors derived from the Autism-spectrum Quotient (AQ; Baron-Cohen et al., 2001), reporting that participants with high levels of social difficulties were faster on the adult EFT than participants with low levels of social difficulty, whereas participants who differed in non-social traits (attention to patterns, interests and details) were comparable in EFT performance. Third, DiCriscio and Troiani (2017) reported that better figure-ground performance (from the Test of Visual Perceptual Skills-Third Edition) in adults was associated with higher scores on the aloof subscale of the Broad Autism Phenotype Questionnaire, but not with scores on the pragmatic language or rigidity subscales. Finally, and importantly, English et al. (2015) reported that reduced LVF bias in spatial attention correlated selectively with higher scores on the social difficulties factor of the AQ for an adult sample.

In summary, there is evidence that the autism spectrum is characterised by both proficiency in local visual processing (in particular, figure disembedding) and reduced LVF bias in spatial attention, and that both phenomena may reflect diminished right hemisphere dominance for visuospatial processing. The major aim of the present study was to investigate whether these two features of visuospatial cognition are linked, and to further test whether each is associated with a social but not with a non-social dimension of autism. This aim was addressed using samples of neurotypical adults selected for extreme scores on autistic trait dimensions rather than by comparing autistic and non-autistic samples. We took this approach for two reasons. First, it is difficult to disentangle the influence of social and non-social dimensions when comparing clinical and non-clinical samples since clinical samples are necessarily extreme on both dimensions. In contrast, social and non-social dimensions are essentially independent in neurotypical populations (English et al., 2020; Russell-Smith et al., 2012) and selecting extreme groups on autistic traits provides greater statistical power than using unselected samples (see Cribb et al., 2016). Second, there is demonstrated continuity across autistic and high autistic trait neurotypical samples in evidence for proficiency in figure disembedding (for reviews, see Cribb et al., 2016; Muth et al., 2014) and a reduced LVF bias (English et al., 2023).

Thus, the present study investigated for the first time associations between figure disembedding proficiency, LVF bias and dissociable dimensions of autistic traits. This was achieved first by selecting groups of young adult participants who were well separated on each of two autistic trait dimensions as assessed by the AQ – social difficulties (SD) and attention to patterns, interests and details (APID), and second by testing for both proficiency in embedded figures search (using the Leuven Embedded Figures Test; L-EFT) and LVF bias (using the greyscales task). Three covariates were also part of the design. Age was included, given evidence of slower EFT search (for reviews, see Capello et al., 2022; Chan & Yan, 2018) and reduced LVF bias (for review, see Jewell & McCourt, 2000) in older adults. Sex differences have typically not been found for the adult EFT (Chan & Yan, 2018), however, Capello et al. (2022) recently reported an advantage for males compared to females on the L-EFT, and Jewell and McCourt (2000) reported a greater LVF bias for males versus females in their line-bisection meta-analysis; accordingly, sex was included as a covariate. The third covariate, hand preference, has been linked to lateralisation of spatial abilities (Vogel et al., 2003), and particularly, there is some evidence of left-handers outperforming right-handers on the EFT (Newland, 1984) and demonstrating more pronounced LVF bias (Jewell & McCourt, 2000).

If proficiency in figure disembedding and LVF bias are associated with the social dimension of autistic traits but not with the non-social dimension, then participants with high levels of SD should demonstrate superior embedded figures performance and reduced LVF bias compared to participants with low levels of SD, whereas participants separated for high and low levels of APID should not differ in embedded figures performance or LVF bias. Furthermore, if enhanced local processing and reduced LVF bias both reflect diminished right hemisphere dominance for visuospatial processing, superior figure disembedding performance should be associated with reduced LVF bias.

## Method

### Participants

This research was approved by the University of Western Australia Human Research Ethics Committee (project RA/4/1/7340). Initially, 704 undergraduate student volunteers (463 females, 237 males, four sex unknown; mean age = 19.91 years; standard deviation = 4.98 years) were screened using the AQ. Next, volunteers with scores in either the lower or upper 33% for AQ SD scores and also scores in either the lower or upper 33% for AQ APID scores were invited to take part in the experimental phase of testing. We selected these groups from the extremes of the trait distributions because of the demonstrated greater power of this design compared to designs that use an unselected sample and either correlations or a median-split (Cribb et al., 2016; Fowler, 1992).

A total of 104 volunteers agreed to participate in the experimental phase. Power for the study was estimated using Monte Carlo simulation as described by Cribb et al. (2016). For N = 104, α = 0.05, population effect size of r = 0.25, and 1,000 samples, power for finding a significant difference between upper and lower 33% groups is estimated to be 0.882.

Descriptive statistics for the participant groups are provided in Table 1. The four groups (High/Low SD × High/Low APID) did not differ significantly in sex ratio as assessed by chi-square contingency analysis (all *p* > 0.92; see Table 1 for frequencies), or in age or Edinburgh Handedness Index (EHI) scores (see Table S1 in Online Supplementary Material (OSM) for distributions) as assessed by between-groups ANOVAs (all *p* > 0.08, all η^2^_p_ < 0.03). As expected, a 2 (SD Group: High vs. Low) × 2 (APID Group: High vs. Low) between-groups ANOVA conducted on the AQ SD scores showed a large effect of SD Group, *F*(1, 100) = 503.95, *MSE* = 6167.09, *p* < 0.001, η^2^_p_ = 0.83, and the corresponding ANOVA on the AQ APID scores showed a large effect of APID Group, *F*(1, 100) = 685.96, *MSE* = 685.96, *p* < 0.001, η^2^_p_ = 0.87, with no other effects significant in either analysis (all *p* > 0.09, all η^2^_p_ < 0.03). Thus, these groups were ideally suited to investigating the independent contributions of social and non-social dimensions of autistic traits to figure disembedding proficiency and LVF bias.

**Table 1:** Descriptive statistics for the four groups selected for low or high scores on AQ Social Difficulties (SD) and low or high scores on AQ Attention to Patterns, Interests and Details (APID)

		Low SD		High SD	
		Low APID	High APID	Low APID	High APID
N (male)		26 (9)	24 (9)	27 (9)	27 (10)
Age (years)	Mean	20.54	20.50	18.81	19.85
	St. Dev	4.51	3.93	1.50	2.84
Handedness Index	Mean	0.57	0.66	0.72	0.86
	St. Dev	0.65	0.52	0.59	0.38
Social Difficulties	Mean	20.50	20.63	36.30	35.67
	St. Dev	2.12	2.65	4.36	4.19
Patterns/Interests/Details	Mean	12.62	21.67	13.59	21.81
	St. Dev	1.79	1.31	1.82	1.71

*St. Dev.* standard deviation

### Materials

#### Questionnaires

The Autism-spectrum Quotient (AQ) questionnaire (Baron-Cohen et al., 2001) is a widely used measure of autistic traits suited to differentiating adults in the general population (Ruzich et al., 2015). Its 50 items describe preferences or patterns of behaviour, and respondents endorse the extent to which each item represents themselves using a four-point scale. We used Austin’s (2005) 1–4 scoring system since it has superior psychometric properties to the original 0–1 system (Stevenson & Hart, 2017). Summed scores for the items loading on the SD and APID factors reported by Russell-Smith et al., (2011; see English et al., 2020, for confirmation of this factor structure) were used to select participants for the four groups.

To assess handedness, the Edinburgh Handedness Index (Oldfield, 1971) was adapted to include a five-point rating scale (always left, mostly left, no preference, mostly right, always right), with the corresponding scores (−2, −1, 0, 1, 2) accumulated and scaled to provide an index ranging from −1 (extreme left-handed) to 1 (extreme right-handed).

#### Greyscales task

This task was taken from English et al. (2017) which was adapted from Nicholls and Roberts (2002). On each trial, the participant was asked to decide which of two horizontal bars – one shaded white-to-black and the other black-to-white – was the darker (see Fig. 1). On half the trials the white-to-black bar was presented above the black-to-white bar and on the remaining trials their positions were swapped. Each bar was 79 pixels in height, and the width of the pair of bars varied across trials, with values of 320, 400, 480, 560, 640, and 720 pixels. For each trial, one bar was shaded slightly darker than the other so there was an objectively correct choice. The darker bar had 100 white pixels replaced with black pixels, and the lighter bar had the opposite colour change for 100 pixels, with altered pixels evenly spread across the bars. This meant that one bar had 0.7–1.6% more black pixels than the other. On each trial, a fixation cross was presented for 1.5 s, followed by the two bars for 5 s, during which the participant was asked to press a key to select the top or bottom bar as the one perceived to be the darker of the two. Speed of responding was not emphasised and if the participant failed to respond within the 5-s window, an identical trial was repeated at the end of the task. There were 24 practice and 120 test trials across which the different stimulus presentation factors were counter-balanced. The key measure of LVF bias was the percentage of test trials on which the participant selected the bar that was shaded darker on the left. A second measure, the percentage of test trials for which the correct (i.e., darker) bar was chosen, checked for the extent to which participants performed the task diligently. The greyscales task was administered using Presentation software (Version 17.0, Neurobehavioral systems).

#### Leuven embedded figures test

**Fig. 2:**
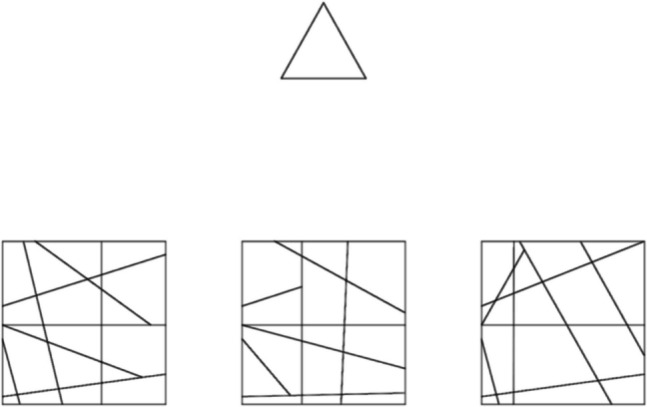
An example of stimuli for the Leuven Embedded Figures Test. In this example, the target is an equilateral triangle and is embedded in the right-most stimulus

The Leuven Embedded Figures Test (L-EFT) was chosen for this study because it is computer-administered (unlike the adult EFT) and has been used to demonstrate superior disembedding for an autistic compared to a non-autistic sample (van der Hallen et al., 2018). Developed by de-Wit and colleagues (2017), the L-EFT includes three practice trials followed by 64 test trials. On each trial, a simple target shape, selected from a set of 16, was presented above three more complex figures arranged horizontally, one containing the target shape (see Fig. 2). Following de-Wit et al., the participant had 3 s to indicate which complex figure contained the target, using three horizontally aligned keys. The screen was cleared for 2.5 s following a response or the elapse of the 3-s response period. Given the short deadline for responding, the key measure of disembedding performance is the percentage of trials answered correctly. The L-EFT was presented using Inquisit 5 Lab (Version 5.0.5.0).

### General procedure

Each participant in the experimental phase was tested individually in a small room, seated 70 cm from a 23-in. LCD monitor running at a 1,920 × 1,080 resolution. Participants completed the handedness questionnaire at the beginning of the session, followed by the two experimental tasks presented in counterbalanced order across participants.

### Data analysis

Each measure from the L-EFT and Greyscales task was subjected to a 2 (SD Group) × 2 (APID Group) ANCOVA, using the covariates age, sex and handedness scores. For brevity, effects for the covariates and their interactions with the Group factors are reported only when significant (*p* < 0.05).

## Results

### Greyscales task performance

Mean accuracy (55.11%) was significantly above chance, *t*(103) = 8.28, *p* < 0.001, *d* = 0.81, indicating that participants were able to perform the task. The ANCOVA conducted on the accuracy scores revealed no significant effects for the SD Group and APID Group factors (largest *F*(1,94) = 0.39.* p* = 0.533, η_p_^2^ = 0.004), indicating that all groups showed similar levels of attention in making the greyscales judgements.

**Fig. 3:**
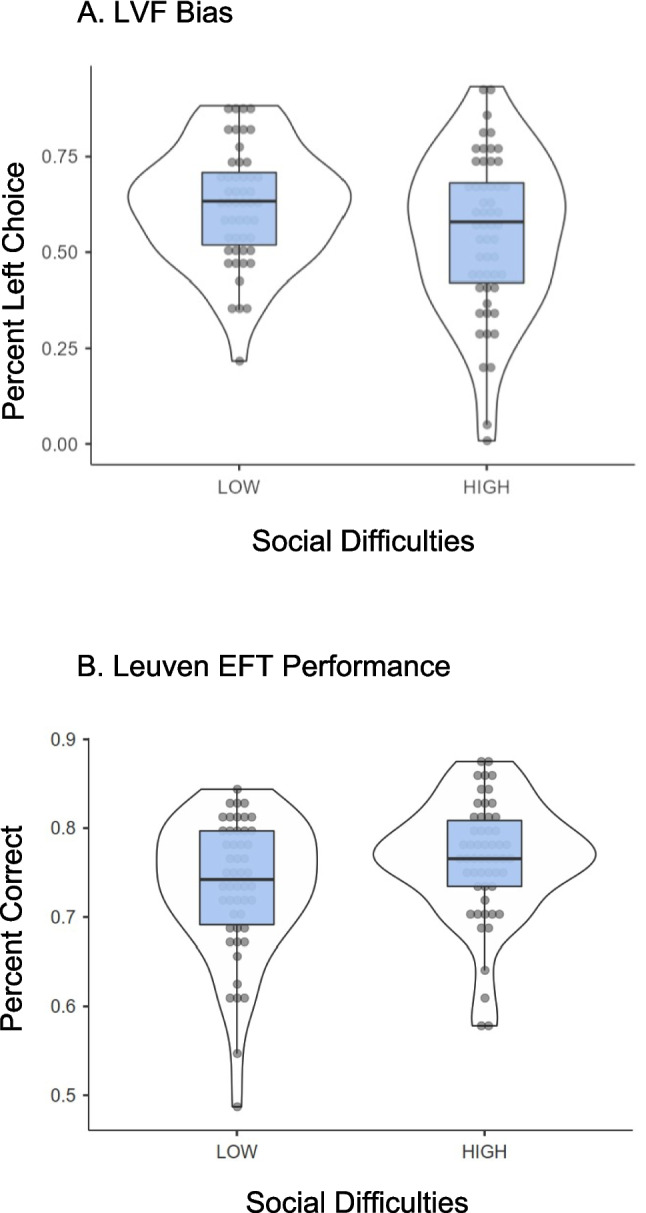
Distributions of scores for left-visual-field bias from the greyscales task (Panel **A**) and accuracy from the Leuven Embedded Figures Test (Panel **B**) as a function of Social Difficulties Group

The key measure of LVF bias, the percentage of trials for which the participant selected the bar shaded darker on the left, was also submitted to ANCOVA with the SD Group and APID Group factors. Only the main effect of SD Group was significant, *F*(1,94) = 4.54, *p* < 0.05, η_p_^2^ = 0.046 (*F* < 1 for the other Group effects). The distributions of scores in Fig. 3A show reduced selection of the left-shaded bar for High SD participants (M = 54.82%; SE = 2.50%) compared to Low SD participants (M = 62.08%; SE = 2.60%). Indeed, the percentage selections of the left-shaded bar was significantly above chance (50%) for the Low SD group, *t*(49) = 5.63, *p* < 0.001, *d* = 0.80, but not for the High SD group, *t*(53) = 1.71, *p* = 0.093, *d* = 0.23, indicating the presence of LVF bias for the Low SD group but not the High SD group.

### L-EFT performance

The SD Group × APID Group ANCOVA conducted on the L-EFT accuracy scores^1^ yielded only a significant main effect of SD Group, *F*(1,94) = 6.09, *p* < 0.05, η_p_^2^ = 0.061 (*F* < 1 for the other Group effects). The distributions of scores in Fig. 3B show that the High SD participants (M = 76.39%; SE = 0.98%) were more accurate than the Low SD participants (M = 73.40%; SE = 1.02%) at identifying the embedded figure. A corresponding ANCOVA conducted on the L-EFT RT data showed no significant main effects or interaction for the SD Group and APID Group factors (all *F* < 1.15, all *p* > 0.28). This indicates that interpretation of the accuracy results is not compromised by evidence for a speed-accuracy trade-off. Incidentally, age did predict L-EFT RT, *F*(1,94) = 4.15, *p* < 0.05, η_p_^2^ = 0.042, with older participants responding more slowly.

### Correlations

The correlation between the index of LVF bias (percentage of trials for which the left-shaded bar was selected) and L-EFT accuracy was significant, *r* = −0.220, *p* < 0.05, indicating that, across the whole sample, lower levels of LVF bias were related to higher levels of L-EFT accuracy (i.e., to greater local processing proficiency).

To check the relationship between the two autistic-trait dimensions, we correlated the SD and APID scores across the 704 participants in the screened sample. This correlation was non-significant, *r* = 0.023, *p* = 0.538, indicating independence of the social difficulties and attention to patterns, interests and details dimensions. Small correlations between these two factors have been reported previously for both undergraduate (*r* = −0.02,* r* = 0.11) and general population (*r* = 0.12) samples (English et al., 2020; Russell-Smith et al., 2011).

## Discussion

Visuospatial cognition in autism and the broader spectrum is characterized by superior local processing (especially in figure disembedding) and reduced LVF bias, two features that may reflect diminished right hemisphere dominance in visuospatial processing. Further, separate lines of preliminary evidence suggest that superior figure disembedding and reduced LVF bias relate uniquely to a social dimension of autistic traits and not to non-social dimensions. Critically, however, no previous study investigated figure disembedding and LVF bias together, and in relation to social and non-social dimensions of autistic symptoms or traits. The present study addressed these limitations by administering the L-EFT and greyscales task to assess proficiency in figure disembedding and LVF bias in participants recruited into a 2 × 2 design, which contrasted the influence of autistic traits pertaining to social difficulties and attention to patterns, interests and details. Based on previous research, it was predicted that participants with high levels of SD would demonstrate superior embedded figures performance and reduced LVF bias compared to participants with low levels of SD, whereas participants separated for high and low levels of APID would not differ in figure disembedding or LVF bias. Additionally, if enhanced local processing and reduced LVF bias both reflect diminished right hemisphere dominance for visuospatial processing, it was expected that superior figure disembedding performance would be associated with reduced LVF bias.

The results confirmed these predictions. On the greyscales task, participants with high levels of SD did not show a preference to choose the left-shaded bar, whereas a significant preference was observed for participants with low SD levels. Responses on the greyscale task did not vary as a function of APID group membership. This pattern of results is consistent with the negative correlation between frequency of choosing the left-shaded bar and SD trait level reported by English et al. (2015). Thus, using an extreme-groups design, the present study confirms that reduced LVF bias in spatial attention is selectively associated with more pronounced social traits.

Regarding figure disembedding proficiency, L-EFT accuracy was significantly greater for High SD compared to Low SD participants and did not differ as a function of APID group. These results establish relationships for L-EFT performance that parallel those reported by Russell-Smith et al. (2012) for the adult EFT and AQ trait dimensions and by DiCriscio and Troiani (2017) for the figure-ground subtest and subscale scores from the BAPQ. Thus, there is now evidence generalised across different autistic-trait measures and several embedded-figures tasks that proficiency in this form of local processing is associated with more pronounced social traits and not with non-social traits of autism.

Significantly, the present study provides two pieces of direct evidence linking local processing proficiency and LVF bias. First, the same pattern of relationships was observed for embedded-figures performance and the greyscales measure of LVF bias concerning the influence of the social and non-social dimensions of autistic traits. Second, there was a significant negative correlation between embedded-figures accuracy and the greyscales measure of LVF, consistent with a connection between greater local-processing proficiency and reduced LVF bias. This is the first time that these two cognitive features previously linked to autism have been investigated together and found to be associated. Arguably, reduced right hemisphere dominance in visuospatial processing could contribute to both a facility in or preference for local over global processing and reduced LVF bias in visuospatial attention. This position is consistent with separate lines of evidence linking right-hemisphere functioning with both global processing (Flevaris et al., 2010; Hübner & Studer, 2009; Iglesias-Fuster et al., 2014; Weissman & Woldorff, 2005) and LVF bias (Fierro et al., 2000; Fink et al., 2002; Foxe et al., 2003; Siman-Tov et al., 2007).

The link between indicators of reduced right hemisphere activation and SD shown here raises an obvious question: how might right-hemisphere functioning be implicated in autism and linked specifically to social difficulties? One possibility is that reduced right hemisphere activation impacts negatively on pragmatic communication and, consequently, on effective social interaction. Two bodies of research support this possibility. First, autism has been associated with under-activity in the right hemisphere. Using a large fMRI data base (*N* = 1035), Subbaraju et al. (2017) reported lower resting-state cortical activity in several regions of the right hemisphere for autistic adolescents and adults compared to their non-autistic counterparts. Second, there is compelling evidence that the right hemisphere plays a critical role in social interaction, particularly in supporting pragmatic communication, including interpreting social, emotional and contextual cues. In an extensive review, Sabbagh (1999) drew parallels between the pragmatic communicative difficulties experienced by autistic individuals and individuals with right-hemisphere damage. These shared difficulties revolve around understanding the communicative intentions of others, which include understanding prosody, identifying themes in discourse, recognising sarcasm, and understanding humour. Thus, if the right hemisphere is typically dominant in supporting pragmatic communication, but this hemisphere is relatively less active in autism, social difficulties could be a consequence.

The study also investigated three covariates – sex, age and handedness. Only age impacted task performance, with older participants tending to be slower responders on the L-EFT task. While this outcome could reflect greater ‘field dependence’ as adults age (i.e., difficulty inhibiting global processing; Chan & Yan, 2018), an alternative explanation is general slowing in processing speed with advancing age (Salthouse, 1996). The absence of significant effects for sex and handedness could reflect limited power, given the under-representation in the samples of males (36% of the sample) and left-handers (see the distributions of handedness scores in Table S1 (OSM), where negative scores represent favouring the left hand). A more highly powered investigation of possible links between autistic traits, handedness and lateralisation of spatial attention is warranted, especially given the elevated rates of atypical handedness in autistic compared to non-autistic samples (Markou et al., 2017).

Returning to the central results, in summary, this study establishes associations between atypical lateralisation of visuospatial functions, local processing, and a particular dimension of autistic traits representing social difficulties. Further work is needed to investigate whether differences in social cognition (e.g., in face processing) mediate these relationships, whether other dimensions of autistic traits not assessed by the AQ (e.g., atypical sensory sensitivity; English et al., 2021) are associated with local–global processing or LVF bias, and whether direct measures of right-hemisphere activation (e.g., through neuroimaging) can provide evidence of function laterality differences related to dimensions of autistic traits.

## Supplementary Information

Below is the link to the electronic supplementary material.

Supplementary file1 (DOCX 402 KB)

## Data Availability

Datasets described in this study are available upon reasonable request to the corresponding author.

## Footnotes

1 One accuracy score was more than three standard deviations below the mean, and so this score was winsorized for analyses of L-EFT accuracy. Outcomes of statistical tests were unchanged when analyses were repeated without winsorizing this value.

## Code availability

Scripts used to collect the data (i.e., behavioural tasks) are available upon reasonable request to the corresponding author.

## Declarations

### Open practices statement

The data and materials reported in this study are available upon reasonable request to the corresponding author. The experiment was not pre-registered.
