# Supplementary material for: Figure disembedding facility and reduced left visual field bias are linked to the social dimension of autistic traits
Source: Atten Percept Psychophys. 2025 Jun 9;87(5):1801–10. doi: 10.3758/s13414-025-03105-7 (PMC12204901; doi:10.3758/s13414-025-03105-7)
Supplement: Supplementary file 1 — Supplementary file1 (DOCX 402 KB) [file 13414_2025_3105_MOESM1_ESM.docx]

**Supplementary Material for *Figure disembedding facility and reduced left visual field bias are linked to the social dimension of autistic traits***


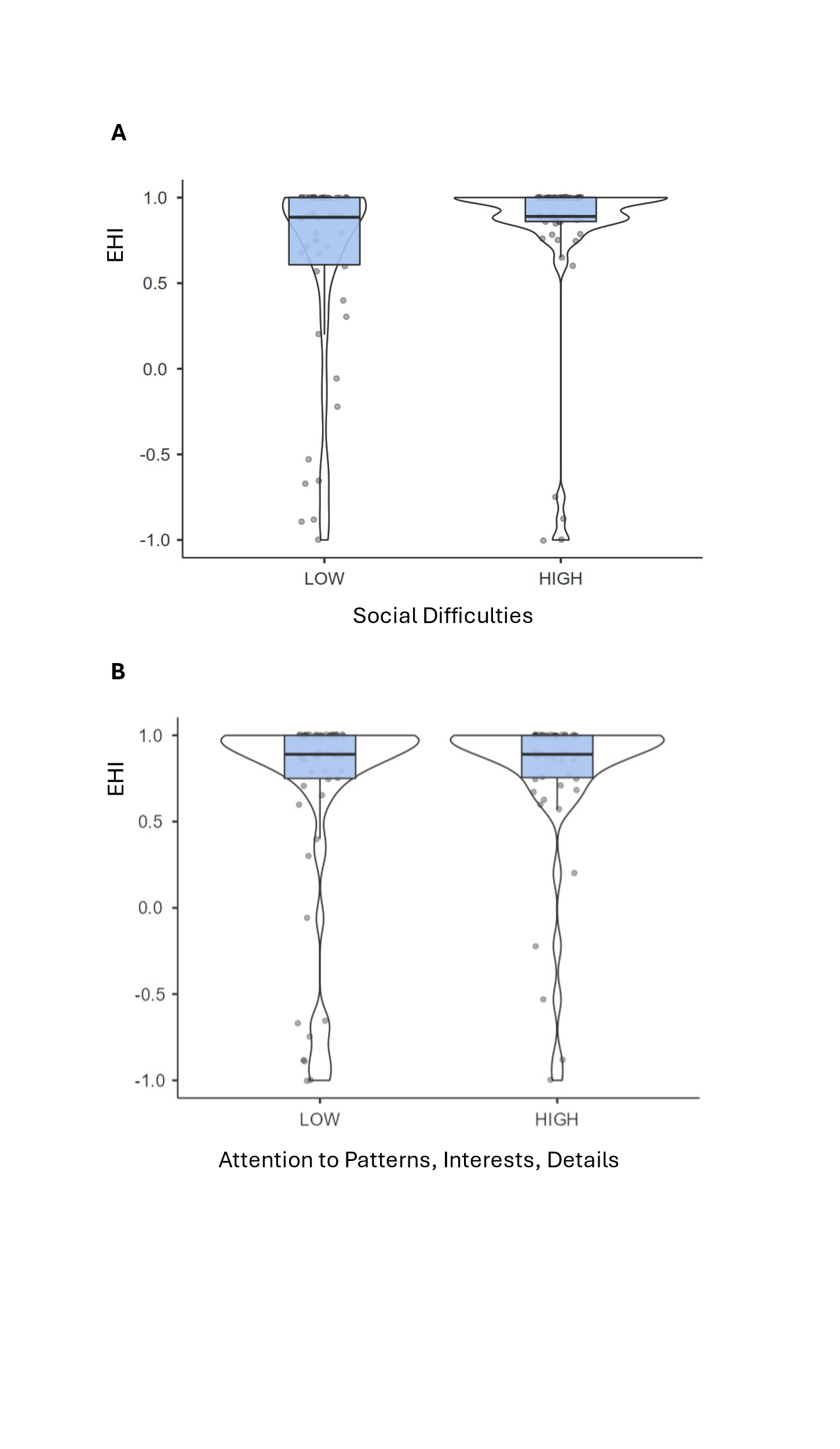


**Figure S1.** Distributions of Edinburgh Handedness Inventory (EHI) scores as a function of Social Difficulties (Panel A) and Attention to Patterns, Interests, and Details (Panel B).
